# Estimation of the Young’s Modulus of Nanometer-Thick Films Using Residual Stress-Driven Bilayer Cantilevers

**DOI:** 10.3390/nano12020265

**Published:** 2022-01-14

**Authors:** Luis A. Velosa-Moncada, Jean-Pierre Raskin, Luz Antonio Aguilera-Cortés, Francisco López-Huerta, Agustín L. Herrera-May

**Affiliations:** 1 Micro and Nanotechnology Research Center, Universidad Veracruzana, Boca del Rio 94294, Mexico; luchoa_23@outlook.com; 2 Institute of Information and Communication Technologies, Electronics and Applied Mathematics (ICTEAM), Université Catholique de Louvain (UCL), 1348 Louvain-la-Neuve, Belgium; jean-pierre.raskin@uclouvain.be; 3 Departamento de Ingeniería Mecánica, DICIS, Universidad de Guanajuato, Salamanca 36885, Mexico; aguilera@ugto.mx; 4 Facultad de Ingeniería Eléctrica y Electrónica, Universidad Veracruzana, Boca del Rio 94294, Mexico; frlopez@uv.mx; 5 Maestría en Ingeniería Aplicada, Facultad de Ingeniería de la Construcción y el Hábitat, Universidad Veracruzana, Boca del Rio 94294, Mexico

**Keywords:** bilayer cantilever, deflections, thin films, residual stresses, young’s modulus

## Abstract

Precise prediction of mechanical behavior of thin films at the nanoscale requires techniques that consider size effects and fabrication-related issues. Here, we propose a test methodology to estimate the Young’s modulus of nanometer-thick films using micromachined bilayer cantilevers. The bilayer cantilevers which comprise a well-known reference layer and a tested film deflect due to the relief of the residual stresses generated during the fabrication process. The mechanical relationship between the measured residual stresses and the corresponding deflections was used to characterize the tested film. Residual stresses and deflections were related using analytical and finite element models that consider intrinsic stress gradients and the use of adherence layers. The proposed methodology was applied to low pressure chemical vapor deposited silicon nitride tested films with thicknesses ranging from 46 nm to 288 nm. The estimated Young’s modulus values varying between 213.9 GPa and 288.3 GPa were consistent with nanoindentation and alternative residual stress-driven techniques. In addition, the dependence of the results on the thickness and the intrinsic stress gradient of the materials was confirmed. The proposed methodology is simple and can be used to characterize diverse materials deposited under different fabrication conditions.

## 1. Introduction

Accurate values of the Young’s modulus are essential to correctly quantify the stiffness of the structures under different loading conditions. A comprehensive understanding of this mechanical property in very thin films is critical to the proper design of small-scale micromachined devices. The Young’s modulus of materials with nanometric dimensions (especially below 100 nm) can vary due to the effect of small size [1] and the fabrication process [2]. Therefore, its correct estimation is an area of great interest in many fields such as microelectronics, protective coatings, and nanoelectromechanical systems (NEMS). At this level, the determination of the Young’s modulus requires very different procedures to the traditional uniaxial tensile tests of bulk samples. Several techniques, including nanoindentation [3,4,5], bulge test [6,7,8], electrostatic pull-in experiments [9,10], or resonant-based methods [11,12] have been developed for this purpose. However, they often require complex experimental setups and extraction procedures that complicate the replicability of the experimental results. The main difficulty of these tests lies in the need to apply external loads that can eventually disturb the samples and produce noise in the measurements.

The residual stresses of materials deposited by surface micromachining processes can be used as a means of actuation to extract the Young’s modulus of thin films without external manipulation of the samples. An example is the on-chip nanomechanical testing laboratory developed to apply a uniaxial load to the tested films using the internal stress present in a well-characterized reference material [13]. The test methodology is simple, which allows the study of the stress-strain response of a wide range of materials. Thus, different properties such as Young’s modulus, yield stress or fracture stress, fracture strain, and strain hardening can be estimated. In addition, this procedure can also be implemented in a semiconductor device production line [14]. Although ultrathin films can be evaluated with this technique, long actuator beams are needed to provide accurate measurements and large strains. This is an important challenge because of the difficulty of fabricating structures with high aspect ratios (length/thickness). Another proposal consists of combining the measurement of the internal stress after film deposition with the measurement of the corresponding internal elastic strain of freestanding beams [15]. This method is simple and easy to implement but is limited to thin films that have high internal stresses. Furthermore, the accuracy of the strain measurement requires long beams that can suffer from stiction in the release steps. Recently, the deflections of residual stress-driven bilayer cantilevers integrated by a tested film and a well-characterized reference layer have been used to study the elastic properties of ultrathin films [16,17]. In these cases, the parameters that are evaluated are both Young’s modulus and mismatch strain of the tested film. Due to these reasons, several cantilevers with different thickness ratios between the tested film and the reference layer must be fabricated. Thus, the intersection of the corresponding stress-deflection curves gives the solution under the assumption that the mismatch strain is the same in all specimens. Accurate measurements can be achieved by applying this method on tested films with thicknesses less than 100 nm due to the large curvatures of the deflected cantilevers. However, the control of thickness in the micromachining process is a critical issue that could limit the reproducibility of the results and hinder the evaluation of materials with high etching rates. Moreover, the presence of intrinsic stress gradients and the use of an adherence layer seriously influence the precision of the results.

In this work, we propose the use of residual stress-driven bilayer cantilevers to estimate the Young’s modulus of thin films with nanometer thicknesses. Our proposal is motivated by the large curvature variations of fully released cantilevers, resulting in increased sensitivity to tested film characteristics. However, we consider wafer-level measurement of residual stresses in both the reference layer and the tested film in comparison to the investigations reported in previous reports [16,17]. The in-situ measurement of these residual stresses allows the characterization of the elastic mismatch strain. Therefore, it is not required to vary the thickness ratio among the fabricated cantilevers, since the Young’s modulus of the tested film is the only parameter to evaluate. Fabricating cantilevers with common thicknesses eliminates the impact that changes in material sizes have on the results and simplifies thickness control in the fabrication process. The tested film is characterized by relating the measured residual stresses to the corresponding deflections. For this, we propose analytical and finite element models from a static analysis of the cantilever. The models consider the effects of intrinsic stress gradients through the thickness of the materials and the possible use of an adhered layer to strengthen the bond between the reference layer and the tested film. The consideration of these effects is relevant for the correct estimation of the elastic properties of the thin films. The results of our methodology are in good agreement with experimental data reported in the literature.

## 2. Estimation Methodology

Bilayer cantilevers deflect when released from their base substrate due to the difference in the residual stresses in the materials used to fabricate them. The magnitude of the deflection depends on the elastic properties of the materials and the intensity of the residual stresses stored during the fabrication process. Therefore, the Young’s modulus of the tested film can be deduced if the mechanical properties of the reference layer are known and both deflections and residual stresses are measured. Occasionally, an additional layer is deposited to promote adhesion between the reference layer and the tested film. In such cases, it is also necessary to know the residual stresses and the mechanical properties of this adherence layer to correctly characterize the tested film. The mechanical relationship between deflections and residual stresses was found through analytical and finite element models. The analytical model was developed from a large deflection analysis of the flexible structure, while the finite element model (FEM) was carried out in the ANSYS^®^ Workbench software using nonlinear static structural analysis.

### 2.1. Analytical Modeling

The relaxation of the residual stresses stored in the materials during the fabrication process causes an internal bending moment that deflects the cantilever to an equilibrium position. The radius of curvature *R* of the deflected cantilever can be related to the internal bending moment *M* from the Euler–Bernoulli beam equation [18,19]:

(1) $$\frac{1}{R}=\frac{M}{{\left(EI\right)}_{e}},$$

where *(EI)_e_* is the equivalent bending rigidity of the cantilever.

**Figure 1:**
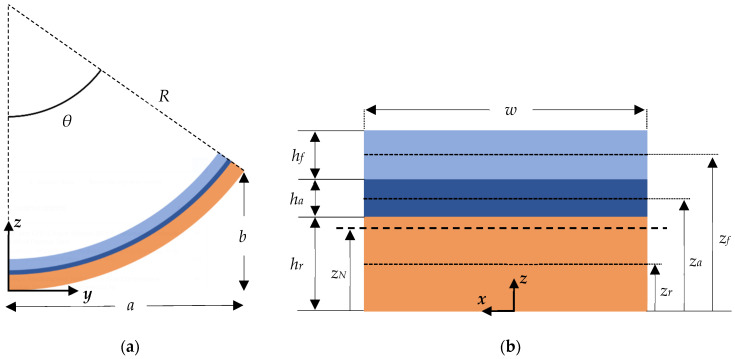
Schematic representation of the residual stress-driven cantilever. (**a**) Deflection profile after released. θ is the angular deflection of the cantilever. (**b**) Geometrical parameters of the cross-section. The reference layer is supposed to be the material located at the bottom of the structure.

The horizontal (*a*) and vertical (*b*) deflections of the cantilever are determined as shown in Figure 1a:

(2) $$a=R\sin\left(\frac{L}{R}\right),$$

(3) $$b=R\left[1-\cos\left(\frac{L}{R}\right)\right],$$

where *L* is the length of the cantilever.

#### 2.1.1. Bending Rigidity of the Cantilever

The equivalent bending rigidity of the cantilever *(EI)_e_* is given as reported in a previous report [20]:

(4) $${\left(EI\right)}_{e}={\bar{E}}_{r}\left[I_{r}+z_{r}A_{r}\left(z_{r}-z_{N}\right)\right]+{\bar{E}}_{f}\left[I_{f}+z_{f}A_{f}\left(z_{f}-z_{N}\right)\right]+{\bar{E}}_{a}\left[I_{a}+z_{a}A_{a}\left(z_{a}-z_{N}\right)\right],$$

where *z_r,f,a_* is the distance between the neutral axis of each material and the bottom of the cantilever. The subscripts *r*, *f*, *a* denote the reference layer, the thin film, and the adherence layer, respectively. In case of homogeneous cross sections, *z_r_*, *z_f_*, and *z_a_* are calculated as shown in Figure 1b:

(5) $$z_{r}=\frac{h_{r}}{2},$$

(6) $$z_{a}=h_{r}+\frac{h_{a}}{2},$$

(7) $$z_{f}=h_{r}+h_{a}+\frac{h_{f}}{2},$$

where *h_r,f,a_* is the thickness of each individual material.

Considering rectangular cross-sections, the cross-sectional area (*A_r,f,a_*) and moment of inertia (*I_r,f,a_*) of the three materials are determined as:

(8) $$A_{r,f,a}=w_{}h_{r,f,a},$$

(9) $$I_{r,f,a}=\frac{1}{12}w_{}h_{r,f,a}^{3},$$

where *w* is the width of the cantilever. The moment of inertia of each individual material is calculated with respect to its own neutral axis, that is, passing through its center of symmetry.

The position of the neutral axis of the entire structure (*z_N_*) is obtained as mentioned in previous reports [20]:

(10) $$z_{N}=\frac{z_{r}{\bar{E}}_{r}A_{r}+z_{f}{\bar{E}}_{f}A_{f}+z_{a}{\bar{E}}_{a}A_{a}}{{\bar{E}}_{r}A_{r}+{\bar{E}}_{f}A_{f}+{\bar{E}}_{a}A_{a}}$$

The biaxial Young’s modulus of each material ($\bar{E}$*_r,f,a_*) is:

(11) $${\bar{E}}_{r,f,a}=\frac{E_{r,f,a}}{1-v_{r,f,a}},$$

where *E_r,f,a_* and *ν_r,f,a_* are the Young’s modulus and the Poisson ratio, respectively.

#### 2.1.2. Internal Bending Moment

**Figure 2:**
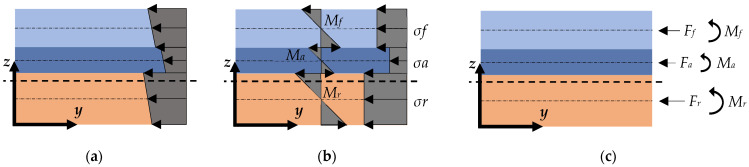
(**a**) Residual stresses stored in the materials during the fabrication process of the cantilever. (**b**) Uniform stresses and intrinsic stress gradients that form the total residual stresses. (**c**) Equivalent system of axial forces and moments.

The residual stresses in each material (Figure 2a) can be divided into a uniform component and an intrinsic stress gradient (Figure 2b). Uniform residual stresses are positive if they cause compression in the materials once the cantilever is released from its base substrate. On the other hand, intrinsic stress gradients are positive if they produce out-of-plane deflection towards the positive *z*-axis. The uniform stress and the intrinsic stress gradient can be represented as an axial force and a moment load, respectively, both acting uniformly over the material cross-section (Figure 2c). By the moment equilibrium around *z_N_*,

(12) $$M=F_{a}\left(z_{a}-z_{N}\right)+F_{f}\left(z_{f}-z_{N}\right)+F_{r}\left(z_{r}-z_{N}\right)+M_{a}+M_{r}+M_{f},$$

where *M* is the internal bending moment that deflects the fully released cantilever. The axial force of each material *F_r,f,a_* expressed in terms of the uniform stress *σ_r,f,a_* is given by:

(13) $$F_{r,f,a}=\sigma_{r,f,a}A_{r,f,a}$$

Generally, *σ_r,f,a_* is determined using the Stoney formula by the measurement of the radius of curvature of the base substrate before and after deposition of each material [21]. Changes in substrate curvatures can be accurately measured using mechanical, capacitive, or optical methods [21]. This technique is quick and practical for the estimation of the uniform residual stresses of wafer-level thin films. However, it has the limitation of being accurate only if the deposited material produces substantial changes in the initial curvature. Curvature changes are practically imperceptible when the thickness of the deposited material is extremely small compared to that of the substrate. In such cases, alternative experimental techniques, such as X-ray diffraction, ultrasound, or Raman spectroscopy should be considered [21,22].

The moment load of the individual materials *M_r,f,a_* can be experimentally estimated by applying Equation (1) in corresponding freestanding monolayer beams. Monolayer beams can be fabricated alongside bilayer cantilevers over the same base substrate following a single fabrication process. Then,

(14) $$M_{r,f,a}=\frac{{\bar{E}}_{r,f,a}I_{r,f,a}}{R_{r,f,a}},$$

where *R_r,f,a_* is the radius of curvature of each monolayer beam. The radii of curvature of the cantilevers (*R* and *R_r,f,a_*) are estimated from the deflection profile using the Taubin method [23]. Nevertheless, Equations (2) and (3) can be used to estimate each radius of curvature in the cases in which only the horizontal or vertical deflections of the cantilever tip are measured.

#### 2.1.3. Solution and Simplification Procedures

The Young’s modulus of the tested film *E_f_* is obtained by numerically solving the system of linear equations composed of Equations (1) and (4)–(14). However, the analytical model can be reduced to a single expression:

(15) $$k=\frac{-6C_{1}+h_{r}{\bar{E}}_{r}C_{2}}{h_{r}{\bar{E}}_{r}\left[m_{a}^{4}\lambda_{a}^{2}+4m_{a}^{3}\lambda_{a}\left(m_{f}\lambda_{f}+1\right)+6m_{a}^{2}\left(m_{f}^{2}\lambda_{a}\lambda_{f}+2m_{f}\lambda_{f}+\lambda_{a}\right)+4m_{a}\left(m_{f}^{3}\lambda_{a}\lambda_{f}+3m_{f}^{2}\lambda_{f}+3m_{f}\lambda_{f}+\lambda_{a}\right)\right]+h_{r}{\bar{E}}_{r}C_{3}},$$

where,

(16) $$C_{1}=\left[m_{a}m_{f}\left(m_{a}+m_{f}\right)\left(\lambda_{f}\sigma_{a}-\lambda_{a}\sigma_{f}\right)\right]+\left[m_{a}\left(m_{a}+1\right)\left(\lambda_{a}\sigma_{r}-\sigma_{a}\right)\right]+\left[m_{f}\left(m_{f}+2m_{a}+1\right)\left(\lambda_{f}\sigma_{r}-\sigma_{f}\right)\right],$$

(17) $$C_{2}=\left(k_{a}m_{a}^{3}\lambda_{a}+k_{f}m_{f}^{3}\lambda_{f}+k_{r}\right)\left(m_{a}\lambda_{a}+m_{f}\lambda_{f}+1\right),$$

(18) $$C_{3}=1+m_{f}\lambda_{f}\left(4+6m_{f}+4m_{f}^{2}\right)+m_{f}^{4}\lambda_{f}^{2},$$

where *λ_f_* = $\bar{E}$*_f_*/$\bar{E}$*_r_* is the biaxial modulus ratio of the tested film and the reference layer, and λ*_a_* = $\bar{E}$*_a_*/$\bar{E}$*_r_* is the biaxial modulus ratio of the adherence layer and the reference layer. *m_f_* = *h_f_/h_r_* is the thickness ratio of the tested film and the reference layer, and *m_a_* = *h_a_/h_r_* is the thickness ratio of the adherence layer and the reference layer. *k* = 1/*R*, *k_r_* = 1/*R_r_*, *k_f_* = 1/*R_f_* and *k_a_* = 1/*R_a_* are defined as the curvature of the bilayer cantilever, reference layer, tested film, and adherence layer, respectively.

The terms related to the adherence layer are overridden in the analytical model if the deposition of this material is not required during the fabrication process. In those cases, Equation (15) can be simplified as:

(19) $$k=\frac{\left[-6m_{f}\left(m_{f}+1\right)\left(\lambda_{f}\sigma_{r}-\sigma_{f}\right)\right]+\left[h_{r}{\bar{E}}_{r}\left(m_{f}\lambda_{f}+1\right)\left(k_{f}m_{f}^{3}\lambda_{f}+k_{r}\right)\right]}{h_{r}{\bar{E}}_{r}\left[1+m_{f}\lambda_{f}\left(4+6m_{f}+4m_{f}^{2}\right)+m_{f}^{4}\lambda_{f}^{2}\right]}$$

Equation (19) can be further simplified if the reference layer and the tested film do not develop intrinsic stress gradients (*M_r,f_* = 0):

(20) $$k=\left[\frac{-6m_{f}\left(\lambda_{f}\sigma_{r}-\sigma_{f}\right)}{h_{r}{\bar{E}}_{r}}\right]\frac{\left(m_{f}+1\right)}{1+m_{f}\lambda_{f}\left(4+6m_{f}+4m_{f}^{2}\right)+m_{f}^{4}\lambda_{f}^{2}}$$

Equation (20) can be also expressed in terms of the uniform residual strain of the reference layer (*e_r_*) and the tested film (*e_f_*) using the Hooke’s law (*σ_r,f_* = *e_r,f_* ${\bar{E}}_{r,f}$):

(21) $$k=\left[\frac{-6\lambda_{f}m_{f}\left(e_{r}-e_{f}\right)}{h_{r}}\right]\frac{\left(m_{f}+1\right)}{1+m_{f}\lambda_{f}\left(4+6m_{f}+4m_{f}^{2}\right)+m_{f}^{4}\lambda_{f}^{2}}$$

This expression is the generalization of the Stoney formula for uniform mismatch strain in the tested film [24]. It was used in the aforementioned bilayer cantilever-based methods to extract the Young modulus of ultrathin films [16,17]. However, Equation (21) can be only used in the cases where the contribution of the intrinsic stress gradients to the internal bending moment is very small compared to that from the uniform stress components.

Finally, if the stiffness of the reference layer is much larger than the tested film (*h_r_* ≫ *h_f_*) such that it does not develop uniform residual stresses (i.e., *σ_r_* = 0), the second part of Equation (20) is simplified, obtaining the classical Stoney formula:

(22) $$k=\frac{6m_{f}\sigma_{f}}{h_{r}{\bar{E}}_{r}}=\frac{6h_{f}\sigma_{f}}{h_{r}^{2}{\bar{E}}_{r}}$$

### 2.2. Finite Element Modeling

**Figure 3:**
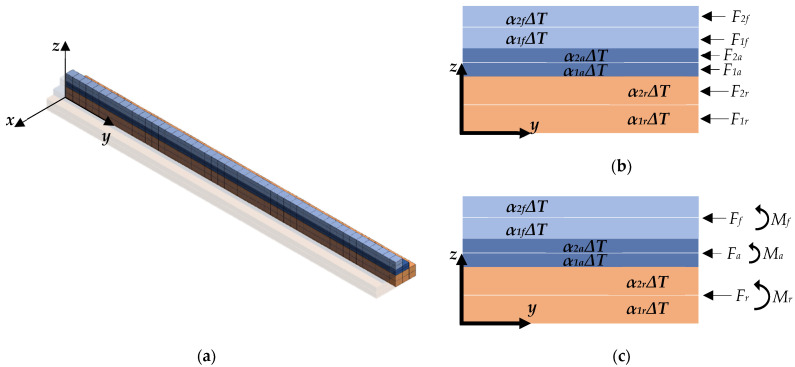
Finite element model of the bilayer cantilever. (**a**) 3D multibody solid meshed with hexagonal elements. (**b**) The thermal strains applied to the two sections of each material produce axial forces due to the interaction between the parts. (**c**) The combined action of the two axial forces in each material produces the uniform stresses and intrinsic stress gradients.

The cantilever is modeled by grouping the materials into a multibody solid that is then fixed at one end (Figure 3a). Each material is split into two symmetrical sections over its thickness. The symmetry of the structure in the *yz*-plane is exploited to simplify the model to half the width. All parts are connected using shared topologies rather than contact regions to have a continuous mesh across the model. The model is meshed with SOLID186 higher-order 3D 20-node hexagonal elements that exhibit quadratic displacement behavior.

Deflections are induced in the model by applying a uniform temperature gradient Δ*T* to the multilayer material of the solid body. The application of the uniform temperature gradient produces thermal strains in the sections according to the following expression:

(23) $$e_{1\left(r,f,a\right)}=\alpha_{1\left(r,f,a\right)}\Delta T,$$

(24) $$e_{2\left(r,f,a\right)}=\alpha_{2\left(r,f,a\right)}\Delta T,$$

where *e_1(r,f,a)_* and *e_2(r,f,a)_* are the thermal strain of the bottom and top sections of each material, respectively. *α_1(r,f,a)_* and *α_2(r,f,a)_* are the specific thermal expansion coefficients of the bottom and top sections of each material, respectively.

Thermal strains produce axial forces in the sections due to the interaction between the parts (Figure 3b). The values of the thermal strains expressed in terms of their respective axial forces are:

(25) $$e_{1\left(r,f,a\right)}=\frac{F_{1\left(r,f,a\right)}}{{\bar{E}}_{r,f,a}A_{r,f,a}},$$

(26) $$e_{2\left(r,f,a\right)}=\frac{F_{2\left(r,f,a\right)}}{{\bar{E}}_{r,f,a}A_{r,f,a}},$$

where *F_1(r,f,a)_* and *F_2(r,f,a)_* are the axial forces in the bottom and top sections of each material, respectively.

The required axial forces are determined by applying the following two boundary conditions to each material. First, the sum of the axial forces of the material must be equal to the equivalent force produced by the uniform residual stress (Figure 3c):

(27) $$F_{1\left(r,f,a\right)}+F_{2\left(r,f,a\right)}=-F_{r,f,a}$$

Second, the sum of the moments around the neutral axis of the material must be equal to the moment load produced by the intrinsic stress gradient (Figure 3c):

(28) $$F_{2\left(r,f,a\right)}-F_{1\left(r,f,a\right)}=\frac{-4M_{r,f,a}}{h_{r,f,a}}$$

The minus sign appears in Equations (27) and (28) due to the relaxation of positive and negative uniform stresses produce compression and tension in the materials, respectively.

Once the values of the thermal strains are estimated using Equations (25)–(28), the thermal expansion coefficients are specified through Equations (23) and (24) by setting the Δ*T* value. Negative thermal expansion coefficients or with a value of zero can be required to perform the simulations correctly. If a material does not develop intrinsic stress gradients, it is not mandatory to split it into two sections, since its respective thermal expansion coefficients will have the same value. The combined action of all axial forces causes the deflection of the cantilever until an equilibrium position is reached.

The Young’s modulus of the tested film (*E_f_*) is found by varying its magnitude in the model until the simulated deflections coincide with those experimentally measured. The simulated deflection profile is extracted from the plane of symmetry of the structure and can be quantified in terms of *a*, *b*, or *R* (Figure 1a).

### 2.3. Accuracy of the Proposed Models

The results of the analytical model may differ from those obtained using FEM if the deflection of the cantilever becomes extremely large. To illustrate this, the deflections of three different residual stress-driven cantilevers (S1, S2, and S3) are considered as examples. The geometric dimensions of the cantilevers, the elastic properties of the materials, the curvatures of the monolayer beams, and the uniform residual stresses are indicated in Table 1 and Table 2. The parameters indicated in Table 1 are common in the examples, whereas the parameters indicated in Table 2 vary according to the cantilever. The uniform residual stress of the tested film is varied in each example to cover a broad range of deflections in the analysis. The cantilevers exhibit similar deflections with the indicated parameters. The examples include the use of adherence layers and the development of intrinsic stress gradients in the materials.

**Figure 4:**
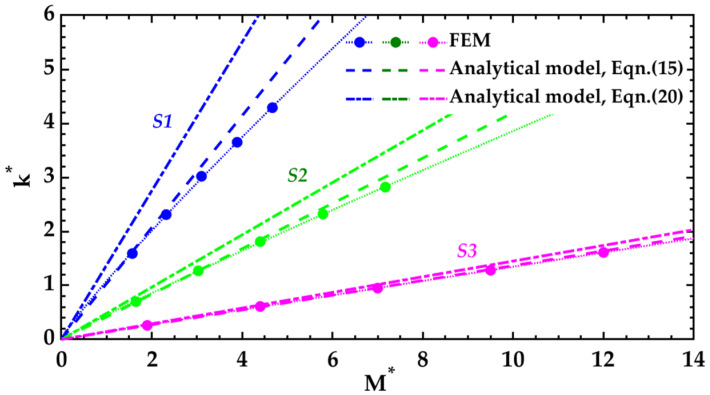
Normalized curvature k* vs. normalized internal bending moment M* for the cantilevers S1, S2, and S3. The dotted curve with circular markers corresponds to the results estimated by FEM. The dashed line represents the results obtained by the analytical model (Equation (15)). The dash-dotted line shows the results obtained by the analytical model if the parameters related to the adherence layer and intrinsic stress gradients are not considered (Equation (20)).

First, the given parameters are used to calculate the deflection of the cantilevers through the proposed models. In these calculations, the Young’s modulus of the tested film has been considered as an input value. The evolution of the normalized curvature (k^*^) with respect the normalized internal bending moment (M^*^) in each cantilever is shown in Figure 4. The expressions for k^*^ and M^*^ are derived following the normalization procedure proposed in previous report [16]:

(29) $$M^{*}=\frac{M}{1\times 10^{-8}\left[N\cdot m\right]}\frac{w^{2}}{{\left(h_{r}+h_{a}+h_{f}\right)}^{2}},$$

(30) $$k^{*}=k\frac{w^{2}}{h_{r}+h_{a}+h_{f}},$$

where *M* and *k* are the internal bending moment and the curvature of each bilayer cantilever, respectively. The analytical model solutions indicate that curvatures are directly proportional to the internal bending moments. However, the results estimated by FEM show that the relationship between the two parameters evolves nonlinearly as the deflections increase beyond their initial values. Nonlinearity can be attributed to stress hardening (which usually appears in structures with very low bending stiffness) and shear deformations. These effects are not considered in the analytical model as it is derived from the Euler-Bernoulli beam theory (Equation (1)). Nevertheless, it is observed that the nonlinear effects drop considerably as the total thickness of the structures increases (results for cantilever S3).

**Figure 5:**
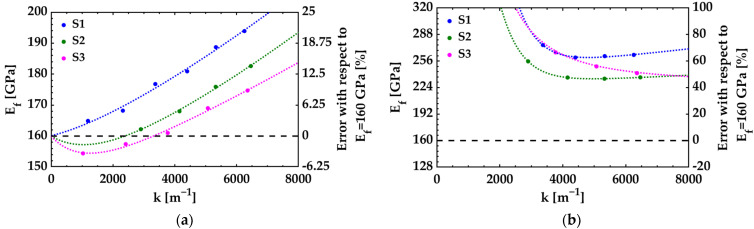
Effect of nonlinear deflections on the estimation of the Young’s modulus of the tested films (*E_f_*). The results were calculated from the curvatures obtained from FEM solutions using: (**a**) Equation (15) and (**b**) Equation (20). The vertical axis on the left side of both graphs represents the results of *E_f_*. The vertical axis on the right side of the graphs represents the error of the results with respect to the real value of *E_f_* (indicated in Table 1).

Nonlinear deflection of cantilevers can have a significant impact on the accuracy of the analytical model. The Young’s modulus of the tested film (*E_f_*) is calculated from the curvatures obtained by FEM using Equation (15). Figure 5a shows the calculated *E_f_* values with respect to the curvatures exhibited in each cantilever (*k*). As expected, the analytical model solutions deviate from the real value of *E_f_* as the deflections increase. However, it is confirmed that the errors produced by nonlinear effects are greater on the cantilevers with smaller thickness (cantilever S1). In other words, for cantilevers with very thin tested films and adherence layers, the accuracy of the analytical model improves with higher *h_r_* values.

The accuracy of the analytical model also decreases when the effects of adherence layers and intrinsic stress gradients are neglected. If the parameters related to these characteristics are not considered in the calculations, Equation (20) can be used to obtain the results. Based on this mathematical expression, the slope of the curves M* versus k* are greater than those obtained using Equation (15) (Figure 4). As a result, the values calculated for the Young’s modulus of the tested films from the curvatures obtained by FEM are unrealistic, especially at small deflections (Figure 5b). A comparative analysis of Equations (15) and (20) shows that the effects of the adherence layer can be omitted in the analyzed examples if *h_a_* is equal to or less than 3 nm, 6 nm, and 3 nm of the cantilevers S1, S2, and S3, respectively. Likewise, it can be determined that the effects of intrinsic stress gradients can be neglected when the relative difference between the internal bending moment (*M*) and the moment produced by the uniform stresses and is less than 2%. However, this last condition is satisfied only if *σ_f_* is greater than 360 MPa, 1300 MPa, and 5100 MPa of the cantilevers S1, S2, and S3, respectively. These values of uniform residual stresses of the tested film are very far from those indicated in Table 2.

It is difficult to establish the validity range of the analytical model proposed in this work due to the large number of variables involved in the equations. However, good results were observed for cantilevers with normalized curvatures below 1.7 (k^*^ ≤ 1.7). This condition is generally fulfilled in cantilevers with angular deflections (Figure 1a) less than ninety degrees (θ < 90°) and with a reference layer of thickness greater than or equal to four hundred nanometers (*h_r_* ≥ 400 nm). Finite element models are generally more accurate because they consider the nonlinear response of the cantilevers when they experience very large deflections. However, some effects that can limit the accuracy of the results are excessive etching of materials and non-homogeneous deposition of the thin films. Furthermore, it is important to note that the models consider initially straight cantilevers with linearly elastic and inextensible materials.

## 3. Results and Discussions

We use the methodology proposed in this work to estimate the Young’s modulus of previously reported silicon nitride (Si_3_N_4_) tested films in two different cases. In the first case, the bilayer cantilever comprises a reference layer of silicon oxide (SiO_2_) [25]. In the second case, the reference layer is made of silicon (Si) [16]. Originally, the research reported by Laconte et al. in [25] aimed to estimate the residual stresses generated in materials during the fabrication process. On the other hand, in the report reported by Favache et al. [16], the bilayer cantilevers were also used to determine the Young’s modulus of Si_3_N_4_ by applying a different procedure. In both reports, the samples were fabricated in the WINFAB (Wallonia Infrastructure Nano Fabrication) cleanroom facilities at Université catholique de Louvain, Louvain-la-Neuve, Belgium (https://sites.uclouvain.be/winfab/NEW_website/. Retrieved 30 November 2021) under similar conditions using different substrates. The results are validated with those previously obtained in the same laboratory using alternative techniques.

### 3.1. Case 1: SiO_2_/Si_3_N_4_ Bilayer Cantilever

**Figure 6:**
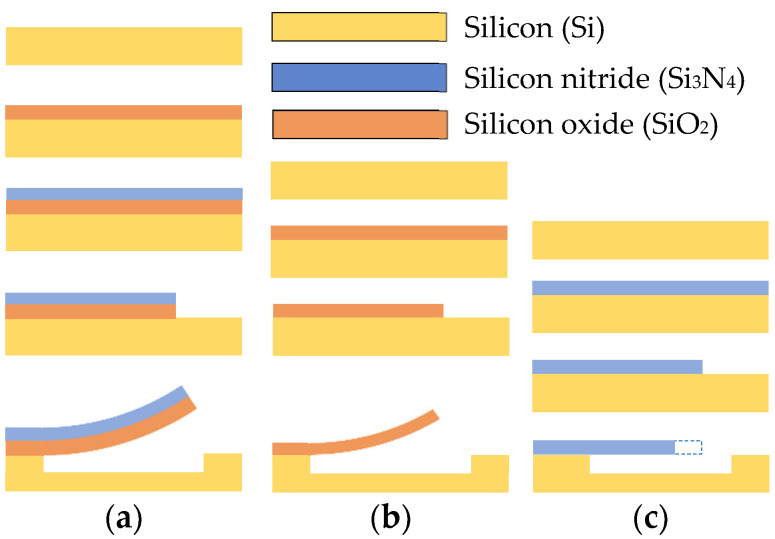
Schematic representation of the main fabrication steps for the case 1. (**a**) SiO_2_/Si_3_N_4_ bilayer cantilever: thermal growth of the SiO_2_ layer, deposition of the Si_3_N_4_ layer, patterning of the deposited layers and release from the Si substrate. (**b**) SiO_2_ beam: growth and patterning of the SiO_2_ layer and release from the Si substrate. (**c**) Si_3_N_4_ beam: deposition and patterning of the Si_3_N_4_ film and release from the Si substrate.

The fabrication process of the SiO2/Si_3_N_4_ bilayer cantilever started with the growth of a thermal SiO_2_ layer on a silicon substrate at 1000 °C under a mixed O_2_/H_2_ atmosphere. Afterward, Si_3_N_4_ was deposited over the thermal SiO_2_ at 800 °C by low pressure chemical vapor deposition (LPCVD) with a stoichiometric mixture of dichlorosilane with an ammonia (SiH_2_Cl_2_/NH_3_) ratio of 1:3. Individual layers of SiO_2_ and Si_3_N_4_ were separately deposited on two different silicon wafers to obtain the monolayer beams. After growing the thin layers, the structures were defined by photolithography and patterned using a plasma etching for the silicon nitride and hydrofluoric acid (HF) for the thermal silicon oxide. The thick silicon wafers were etched in 20% tetramethyl ammonium hydroxide (TMAH) solution at 90 °C for one hour to release the cantilevers and then rinsed in de-ionized water and dried in methanol to avoid structural damage. The deposition of an adherence layer was not considered in the fabrication process because the materials showed high adhesion. A schematic diagram of the fabrication steps is shown in Figure 6.

**Figure 7:**
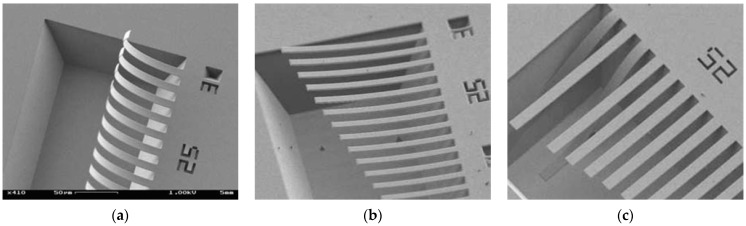
SEM views of the released cantilevers in case 1 (longest showed cantilevers are 150 μm long). (**a**) SiO_2_/Si_3_N_4_ Bilayer Cantilever. (**b**) SiO_2_ beam. Deflection reveals the presence of strain gradients over film thickness. (**c**) Si_3_N_4_ beam. Some stiction appeared after rinsing with water and drying in methanol. Reprinted with permission from [25]. Copyright©2006, Springer Nature.

Corresponding uniform components of the residual stresses were measured using the Stoney formula by wafer curvature measurements [21]. The thicknesses of the deposited materials were verified by ellipsometry while the in-plane dimensions of the cantilevers were measured by SEM. Figure 7 shows the SEM views of the fabricated cantilevers after being released from the Si substrate. The vertical deflection for 100 μm length and 10 μm wide cantilevers was measured using an optical microscope comparing the focus on both ends.

Monolayer SiO_2_ beams exhibited a notable vertical deflection that revealed the presence of intrinsic stress gradients in this material (Figure 7b). In contrast, the tested film appears to be free of the intrinsic stress gradients as their respective freestanding beams remained straight after being released (Figure 7c). The Young’s modulus and the Poisson ratio of the SiO_2_ reference layer and the Poisson ratio of the tested Si_3_N_4_ material were reported in the literature [25]. The dimensions of the structures, the uniform residual stresses, the vertical deflections of the cantilevers, and the elastic properties of the materials are indicated in Table 3.

**Figure 8:**
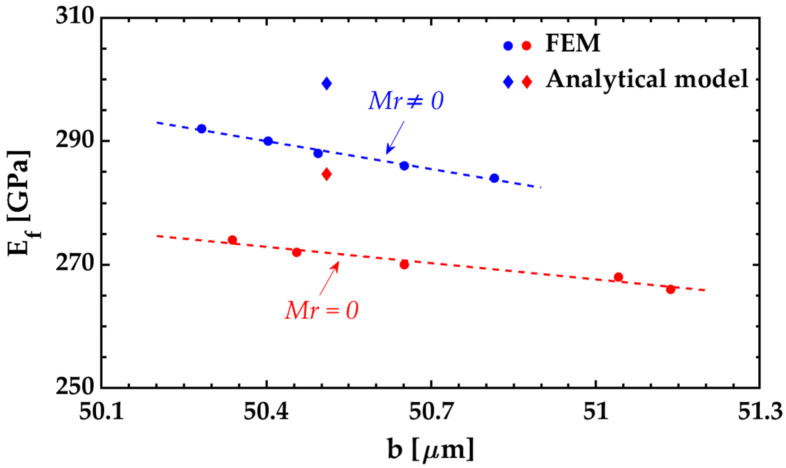
Estimated Young’s modulus of the Si_3_N_4_ tested films for case 1 (SiO_2_/Si_3_N_4_ bilayer cantilever). The colored dots indicate the FEM results for different values of *E_f_* from which linear fits (dashed lines) are made. The correct value of *E_f_* is found by evaluating the measured vertical deflection on the fitted linear function. The incidence of the intrinsic gradient of the SiO_2_ layer on the results is evidenced in the lower values of the Young’s modulus of the Si_3_N_4_ when it is assumed that *M_r_* = 0.

The analytical solution was estimated using Equation (19) since the SiO_2_ layer developed intrinsic stress gradients during the fabrication process. The radii of curvature of the bilayer cantilever and the monolayer beams were calculated from their respective vertical deflections using Equation (3). For the FEM solution, the 3D model was meshed with 250 elements over the length, 15 elements over the half width, 1 element over the thickness of the two sections of the SiO_2_ layer, and 1 element over the thickness of the Si_3_N_4_ film. The silicon nitride tested film was not split into two symmetrical sections since it was free of intrinsic stress gradients. Several simulations were conducted varying the *E_f_* value from 284 to 292 GPa to obtain different Young’s Modulus versus deflection responses. Subsequently, a linear regression was performed on the recorded data to approximate the relationship between the two variables (Figure 8). Then, the correct value of *E_f_* was obtained by evaluating the experimentally measured bilayer cantilever deflection on the fitted linear function. Both silicon oxide and silicon nitride were considered isotropic materials [25].

The Young’s modulus values of the 288 nm thick Si_3_N_4_ tested films found through the models proposed in this work are listed in Table 4. The results of the analytical model agree well with those obtained by FEM. The incidence of the intrinsic gradient of the SiO_2_ layer has on the results was studied by repeating the calculations with *M_r_* = 0. The elimination of *M_r_* leads to an underestimation of 4.9 and 5.7% in the *E_f_* values estimated by the analytical and FEM models, respectively.

The results of the Young’s modulus of the Si_3_N_4_ films for case 1 are slightly above the upper range of the reported values for the same lab (summarized in Table 5). The lack of resolution in the optical microscope and the ineffectiveness of the strategy used to estimate vertical deflection may be the reason. Furthermore, edge effects at the free end of the cantilevers can cause miscalculation of the respective radii of curvature. Nevertheless, it is worth mentioning that Young’s modulus of the silicon nitride can vary from 193 GPa to 338.5 GPa according to the review of existing data reported in the previous report [16].

### 3.2. Case 2: Si/Si_3_N_4_ Bilayer Cantilever

**Figure 9:**
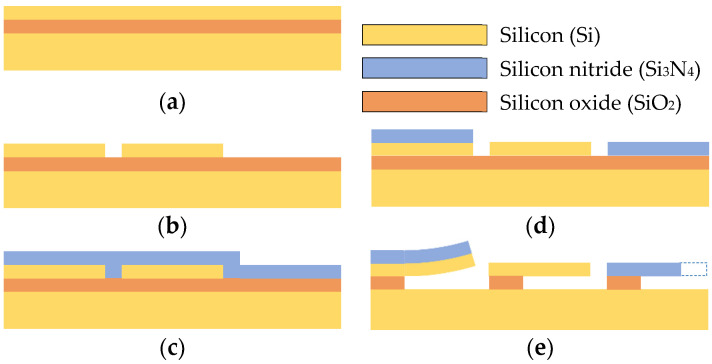
Schematic representation of the main fabrication steps for the case 2 (Si/Si_3_N_4_ bilayer cantilever). (**a**) SOI wafer; (**b**) patterning of the top Si; (**c**) deposition of Si_3_N_4_; (**d**) patterning of Si_3_N_4_; (**e**) etching of the SiO_2_ sacrificial layer.

In this case, the structures were fabricated on a silicon-on-insulator (SOI) wafer following the process shown in Figure 9. The first step was the patterning of the upper Si layer of the SOI wafer by reactive ion etching (RIE) with a sulfur hexafluoride (SF_6_)-based plasma. The Si_3_N_4_ film was then deposited at 790 °C through LPCVD and then patterned by RIE using a mixture of sulfur hexafluoride and silicon tetrachloride (SF_6_/SiCl_4_)-based plasma. At this point, the wafer was cut into four samples (G1, G2, G3, and G4) before releasing the structures by the etching of the SiO_2_ sacrificial layer using HF (73 vol.%). Since HF also etches Si_3_N_4_ at a slower rate than SiO_2_, the release time was varied in each sample to expect obtain bilayer cantilevers with different tested film thicknesses *h_f_* (Table 6). The fabrication process allows the production of Si/Si_3_N_4_ bilayer cantilevers and freestanding monolayer Si and Si_3_N_4_ beams of several lengths.

**Figure 10:**
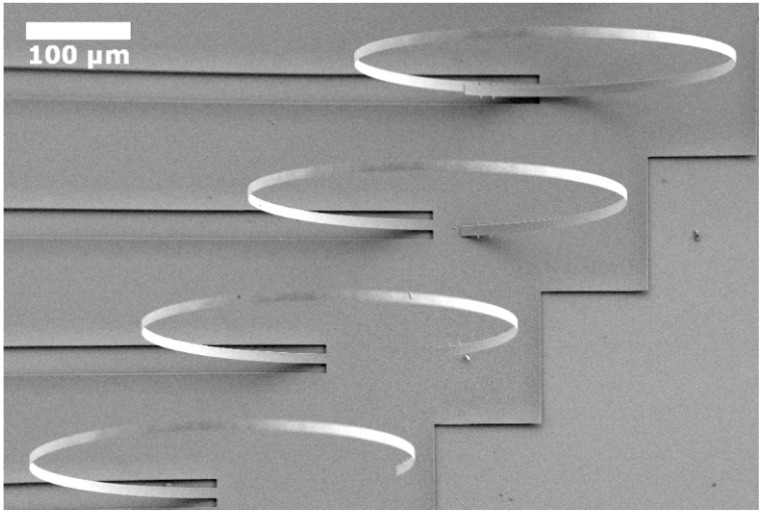
SEM view of the fully released Si/Si_3_N_4_ (sample G1) bilayer cantilevers. Reprinted with permission from [16]. Copyright©2016, AIP Publishing.

The thickness of the Si reference layer was obtained from the SOI wafer specifications, whereas the thickness of the Si_3_N_4_ films was measured by ellipsometry using the Cauchi model [26]. On the other hand, the deflection profile shown by the bilayer cantilevers after being released (Figure 10) was measured using SEM and interferometry. Subsequently, the respective radii of curvature were estimated by interpolating a circle on the deformed shape applying the Taubin method [23].

Freestanding monolayer Si and Si_3_N_4_ beams did not exhibit perceptible deflections, indicating the absence of intrinsic stress gradients. Therefore, the in-plane deformations were used to estimate the uniform residual strains. The residual strains of the Si_3_N_4_ and Si beams were *e_f_* = 0.0032 and *e_r_* ≈ 0 (indicating the absence of uniform residual stresses at the top of the Si layer of the SOI wafer), respectively. The measured thicknesses and curvatures of the Si/Si_3_N_4_ bilayer cantilevers are given in Table 6, while the geometrical and elastic properties required in the models are indicated in Table 7.

**Figure 11:**
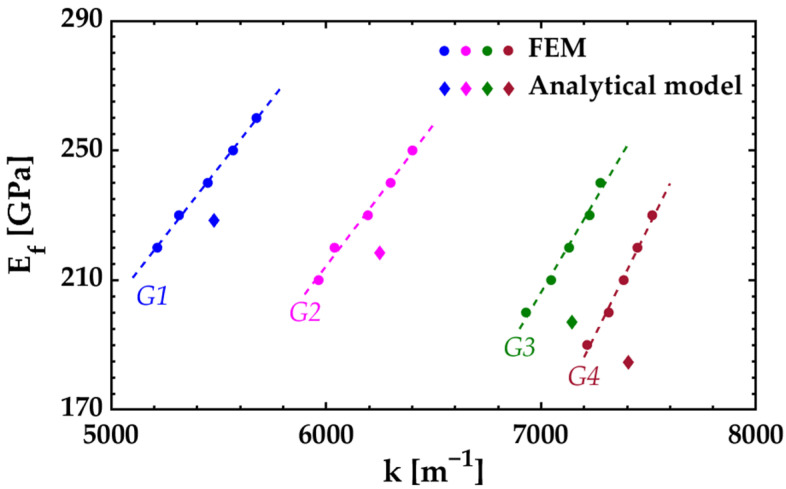
Estimated Young’s modulus of the Si_3_N_4_ tested films for case 2 (Si/Si_3_N_4_ bilayer cantilever).

The analytical solution was estimated from the uniform residual strains using Equation (21) since the materials were free of intrinsic stress gradients. The FEM solution was found following the same extraction methodology of case 1 (Figure 11). The model was meshed with 160 elements over the length, 15 elements over the half width, and 1 element over the thickness of the Si layer and the Si_3_N_4_ film. The materials were not split into two symmetrical sections as they did not develop intrinsic stress gradients during the fabrication process. Experimentally, it was observed that the radius of curvature does not have significant changes in the bilayer cantilevers with lengths ranging between 100 μm and 1.9 mm. Therefore, a length of 200 μm is appropriate to perform the simulations with better results. The thermal expansion coefficients of the materials were calculated from the uniform residual strains using Equations (23) or (24). The simulated deflection profile was extracted in the range of 10 μm to 190 μm across the long axis to avoid edge effects.

In the solutions, Si_3_N_4_ was considered isotropic while Si was considered orthotropic [16]. According to the global coordinate system of Figure 3, the elastic constants of the orthotropic silicon are [27]:

(31) $$\begin{array}{l} \begin{array}{cc} \left[E_{x},E_{y},E_{z}\right]=\left[169,169,130\right] & \left[\mathrm{GPa}\right] \end{array}\\ \left[v_{xy},v_{yz},v_{zx}\right]=\left[0.064,0.36,0.28\right]\\ \begin{array}{cc} \left[G_{xy},G_{yz},G_{zx}\right]=\left[50.9,79.6,79.6\right] & \left[\mathrm{GPa}\right] \end{array} \end{array}$$

where *E*, *v,* and *G* refer to Young’s modulus, Poisson’s ratio, and shear modulus, respectively. For the analytical solution, the biaxial Young’s modulus of silicon was taken from the elastic constants in the *xy*–plane:

(32) $$\begin{array}{cc} {\bar{E}}_{r}=\frac{E_{x}}{1-v_{xy}}=\frac{E_{y}}{1-v_{xy}}=180.55 & \left[\mathrm{MPa}\right] \end{array}$$

Table 8 presents the Young’s modulus values of the Si_3_N_4_ tested films for each of the four samples. The results are in good agreement with those values obtained in silicon nitride films fabricated under similar experimental conditions (Table 5).

The values obtained using the analytical models agree well with those obtained by FEM, but the relative difference between them increases with higher tested film thickness *h_f_*. The increase of *h_f_* has more incidence on the internal bending moment *M* than on the bending rigidity *(EI)_e_* due to the high magnitude of *σ_f_*. Nonproportional increase in the values of *M* and *(EI)_e_* results in larger deflections of the bilayer cantilever. Under large deflections, the curvature *k* does not vary linearly with the internal bending moment *M* and the accuracy of the analytical model decreases (as explained in Section 2.3).

**Figure 12:**
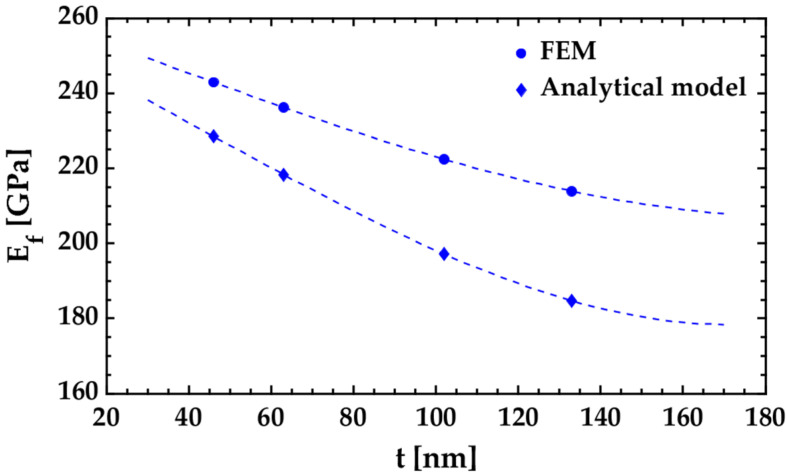
Young’s modulus *E_f_* vs. thickness *h_f_* for case 2 (Si/Si_3_N_4_ bilayer cantilever).

The dependence of the results on the size of the tested film is seen in the *E_f_* versus *h_f_* plot shown in Figure 12. Specifically, it is noticed that the Young’s modulus of the Si_3_N_4_ tested film increases as its thickness decreases. The estimated Young’s modulus of Si_3_N_4_ tested film with a thickness of 40 nm (sample G1) is about 14% higher than that of Si_3_N_4_ tested film with a thickness of 133 nm (sample G4). Nevertheless, it should be considered that the estimation of the Young’s modulus of ultrathin layered materials can be influenced by defects in the test material or by internal factors such as roughness. The study of the influence of these internal parameters on the results is not part of the objectives of this work.

Finally, it was found that the Si_3_N_4_ films tested in case 1 (SiO_2_/Si_3_N_4_ bilayer cantilever) have a higher Young’s modulus than Si_3_N_4_ films tested in case 2 (Si/Si_3_N_4_ bilayer cantilever). This may be because the structural properties of the Si_3_N_4_ deposited on SiO_2_ can be different from the structural properties of the Si_3_N_4_ deposited on Si [25]. In addition, some specific parameters of the fabrication process, such as deposition time, etching time, or etch solutions (which are different in the two investigated cases) can alter the properties of the tested films.

## 4. Conclusions

A methodology to predict the Young’s modulus of nanometer-thick films using residual stress-driven bilayer cantilevers was reported. The bilayer cantilever consists of a well-known reference layer and a tested film that store residual stresses during the fabrication process. The fully released cantilever deflects due to the difference in the residual stresses of the two materials. The measured deflections and residual stresses are related using analytical or finite element models to calculate the Young’s modulus of the tested film. The proposed models include the intrinsic stress gradients and the use of adherence layers. Our methodology was applied to previously reported silicon nitride (Si_3_N_4_)-tested films deposited on silicon oxide (SiO_2_) and silicon (Si) reference layers. The estimated Young’s modulus for the 288 nm thick Si_3_N_4_ tested films deposited on SiO_2_ was 288.3 GPa. On the other hand, the estimated Young’s modulus for the Si_3_N_4_ tested films deposited on Si with thicknesses ranging between 43 and 133 nm varied from 242.9 GPa to 213.9 GPa. The results obtained in this work were in good agreement with reported literature data of Si_3_N_4_ films fabricated under similar conditions. This methodology can be easily used for thin films of different materials. However, it is limited by the resolution of the techniques employed to estimate the residual stresses, deflections, and in-plane dimensions. Future research will focus on estimating the Young’s modulus of thin films using residual stress-driven structures but reducing the dependence of the methodology on the Poisson’s ratio of the tested film and the elastic properties of the reference layer.

## Figures and Tables

**Table 1:** Common parameters in the analyzed examples.

Parameter	Value
Cantilever width, *w*	12 μm
Cantilever length, *L*	150 μm
Thickness of the adherence layer, *h_a_*	30 nm
Thickness of the tested film, *h_f_*	60 nm
Biaxial Young’s modulus of the reference layer, $\bar{E}$*_r_*	250 GPa
Biaxial Young’s modulus of the adherence layer, $\bar{E}$*_a_*	125 GPa
Young’s modulus of the tested film, *E_f_*	160 GPa
Poisson ratio of the tested film, *v_f_*	0.2
Curvature of the reference layer beam, *k_r_*	−890 m^−1^
Curvature of the adherence layer beam, *k_a_*	0
Curvature of the tested film beam, *k_f_*	1790 m^−1^
Uniform residual stress of the reference layer, *σ_r_*	−50 MPa
Uniform residual stress of the adherence layer, *σ_a_*	0

**Table 2:** Parameters for cantilevers S1, S2 and S3.

Parameter	S1	S2	S3
Thickness of the reference layer, *h_r_* [nm]	120	240	480
Uniform residual stress of the tested film, *σ_f_* [MPa]	40–200	100–500	300–1500

**Table 3:** Parameters for case 1 (SiO_2_/Si_3_N_4_ bilayer cantilever).

Parameter	Value	Measurement Technique
Cantilever width, *w*	10 μm	SEM
Cantilever length, *L*	100 μm	SEM
Thickness SiO_2_ layer, *h_r_*	433 nm	Ellipsometry
Thickness Si_3_N_4_ film, *h_f_*	288 nm	Ellipsometry
Vertical deflection bilayer cantilever, *b*	50.51 μm	Optical microscopy
Vertical deflection SiO_2_ beam, *b_r_*	12 μm	Optical microscopy
Vertical deflection Si_3_N_4_ beam, *b_f_*	≈0	Optical microscopy
Young’s modulus SiO_2_, *E_r_*	70 GPa	Reported in [25]
Poisson ratio SiO_2_, *v_r_*	0.2	Reported in [25]
Poisson ratio Si_3_N_4_, *v_f_*	0.27	Reported in [25]
Uniform residual stress SiO_2_ layer, *σ_r_*	−281 MPa	Wafer curvature measurement and Stoney formula
Uniform residual stress Si_3_N_4_ film, *σ_f_*	914 MPa	Wafer curvature measurement and Stoney formula

**Table 4:** Estimated values of the Young’s modulus of the Si_3_N_4_ tested films for case 1 (SiO_2_/Si_3_N_4_ bilayer cantilever).

Result	Analytical Model	FEM	Relative Difference
*E_f_* [GPa]	299.3 ^1^	288.3	3.8%
*E_f_* (*M_r_ =* 0) [GPa]	284.7 ^2^	271.9	4.7%

^1^ Equation (19). ^2^ Equation (20).

**Table 5:** Young’s modulus of silicon nitride thin films deposited in the WINFAB laboratory.

Method	Thickness (nm)	Value (GPa)	Reference
Nanoindentation	250	235 ± 10	[13]
Stoney and freestanding beams	301	233	[15]
Nanoindentation	301	241	[15]
Bilayer cantilever	55	270 ± 20	[16]
This work (case 1)	288	288.3	–
This work (case 2)	46	242.9	–
	63	236.2	
	102	222.4	–
	133	213.9	–

**Table 6:** Thicknesses and Curvatures of the Si/Si_3_N_4_ bilayer cantilevers.

Dimension	G1	G2	G3	G4
Thickness Si layer [nm], *h_r_*	400	400	400	400
Thickness Si_3_N_4_ film [nm], *h_f_*	46	63	102	133
Curvature bilayer cantilever [m^−1^], *k*	5479	6250	7143	7407

**Table 7:** Dimensions and elastic properties for case 2 (Si/Si_3_N_4_ bilayer cantilever).

Parameter	Value	Measurement Technique
Cantilever width, *w*	10 μm	Optical microscopy
Cantilever length, *L*	200 μm	Optical microscopy
Poisson ratio Si_3_N_4_ film, *v_f_*	0.27	reported in [16]
Uniform residual strain Si layer, *e_r_*	≈0	SEM
Uniform residual strain Si_3_N_4_ film, *e_f_*	0.0032	SEM

**Table 8:** Estimated values of the Young’s modulus of the Si_3_N_4_ tested films for case 2 (Si/Si_3_N_4_ bilayer cantilever).

**Sample**	**Analytical Model [GPa] ^1^**	**FEM [GPa]**	**Relative Difference**
G1	228.5	242.9	5.9%
G2	218.3	236.2	7.6%
G3	197.1	222.4	11.4%
G4	184.7	213.9	13.7%

^1^ Equation (21).

## Data Availability

Data is contained within the article.
